# Hybrid Polypyrrole and Polydopamine Nanosheets for Precise Raman/Photoacoustic Imaging and Photothermal Therapy

**DOI:** 10.1002/adhm.202301148

**Published:** 2023-06-20

**Authors:** Hongya Geng, Emily J. Lupton, Yun Ma, Rujie Sun, Christopher L. Grigsby, Giulia Brachi, Xiaorui Li, Kun Zhou, Daniel J. Stuckey, Molly M. Stevens

**Affiliations:** ^1^ Department of Materials Department of Bioengineering Institute of Biomedical Engineering Imperial College London London SW7 2AZ UK; ^2^ Department of Medical Biochemistry and Biophysics Karolinska Institutet Stockholm Stockholm 171 11 Sweden; ^3^ Tsinghua Shenzhen International Graduate School Tsinghua University Shenzhen 518055 China; ^4^ UCL Centre for Advanced Biomedical Imaging Division of Medicine University College London London WC1E 6DD UK

**Keywords:** conductive nanosheets, photoacoustic imaging, photothermal therapy, polypyrrole, Raman imaging

## Abstract

The development of near‐infrared light responsive conductive polymers provides a useful theranostic platform for malignant tumors by maximizing spatial resolution with deep tissue penetration for diagnosis and photothermal therapy. Herein, the self‐assembly of ultrathin 2D polypyrrole nanosheets utilizing dopamine as a capping agent and a monolayer of octadecylamine as a template is demonstrated. The 2D polypyrrole‐polydopamine nanostructure has tunable size distribution which shows strong absorption in the first and second near‐infrared windows, enabling photoacoustic imaging and photothermal therapy. The hybrid double‐layer is demonstrated to increase Raman intensity for 3D Raman imaging (up to two orders of magnitude enhancement and spatial resolution up to 1 µm). The acidic environment drives reversible doping of polypyrrole, which can be detected by Raman spectroscopy. The combined properties of the nanosheets can substantially enhance performance in dual‐mode Raman and photoacoustic guided photothermal therapy, as shown by the 69% light to heat conversion efficiency and higher cytotoxicity against cancer spheroids. These pH‐responsive features highlight the potential of 2D conductive polymers for applications in accurate, highly efficient theranostics.

## Graphical abstract

2D polypyrrole/polydopamine nanosheets are self‐assembled and exhibit enhanced photothermal conversion efficiency and sensitive response to a broad range of pH changes. These nanosheets can be outstanding Raman probes, photoacoustic imaging contrast agents, and used for photothermal therapy.

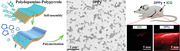

## 1 Introduction

Imaging‐guided therapy using near‐infrared (NIR) light can be a powerful method for cancer treatment.^[^ ^1^ ^]^ Compared to traditional cancer treatments, such as radiotherapy and chemotherapy, photothermal therapy (PTT) is emerging as a supplementary, noninvasive strategy with improved therapeutic outcomes.^[^ ^2^ ^]^ PTT triggers the death of tumour cells through external laser irradiation to induce hyperthermia; it also boasts an easily adjustable dose and has minimal side effects on the surrounding healthy tissues.^[^ ^3^ ^]^ The development of PTT agents with absorption in the NIR window and enhanced photothermal conversion has shown great promise for efficient tumor eradication.^[^ ^4^ ^]^ On the other hand, photoacoustic (PA) imaging can generate 3D images with high spatial resolution and deep tissue penetration of up to 6 cm, overcoming the drawbacks of some conventional techniques, such as autofluorescence and tissue background noise.^[^ ^5^ ^]^ The combination of PA and PTT can provide a promising solution for the diagnosis and treatment of cancer.

Conductive polymers, including polypyrrole (PPy), enable the conversion of light into heat for photothermal ablation therapy and can be used as PA contrast agents with strong NIR absorption beyond 1000 nm.^[^ ^6^ ^]^ Previous studies have shown that the suitability of conductive polymers for PTT and PA imaging depends on their nonradiative thermal deexcitation pathway.^[^ ^7^ ^]^ The Raman spectral signal of conductive polymers such as PPy depends on the protonation states and doping levels.^[^ ^8^ ^]^ These properties endow PPy with the potential ability to probe various redox and acidic/alkaline environments using Raman spectroscopy to obtain the biochemical profiles of cellular and subcellular molecular structures, which compensates for the limited spatial resolution of PA imaging.^[^ ^9^ ^]^ Additionally, the integration of hybrid 2D nanostructures leads to unique optical and electronic properties.^[^ ^8b^ ^]^ This is also a promising alternative to modifying the rigid *π*‐conjugated structures of PPy for on‐demand high photothermal conversion efficiency and dispersibility in aqueous solutions. As a significant component of the natural pigment melanin, polydopamine PD has a molecular structure similar to dihydroxy‐l‐phynylalanine and lysine‐enriched proteins. The high fluorescence quenching and strong NIR absorption make PD a promising agent to form this 2D nanostructure for PTT and PA imaging.^[^ ^10^ ^]^

In this work, we developed 2D theranostic nanosheets consisting of PD and PPy (DPPy) through a facile self‐assembly method. The combination of PD and PPy confers the 2D nanostructures with high biocompatibility, versatility, and stability. We demonstrated an absorbance redshift to the second NIR window and controllable lateral size distribution following self‐assembly with various ratios of pyrrole to PD to offer enhanced photothermal efficiency. The increased NIR‐absorbance also reinforced the contrast and resolution of the PA imaging. Our 2D DPPy structure exhibited Raman signals two orders of magnitude higher than spherical structures. Furthermore, upon exposure to an acidic microenvironment, the reversible protonation and deprotonation of PPy provided 3D subcellular information using Raman microspectroscopy, which highlights the potential of DPPy nanosheets for dual‐mode imaging‐guided tumor PTT.

## 2 Results and Discussion

### 2.1 Construction and Preparation of DPPy Nanosheets

**Figure 1:**
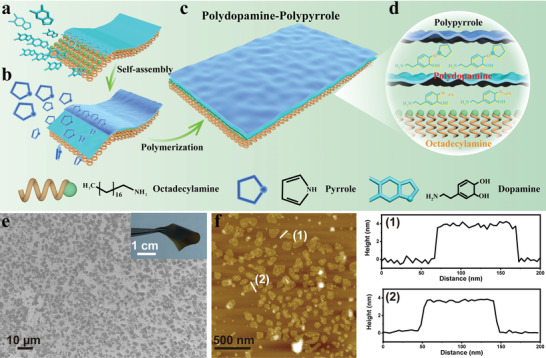
Self‐assembly and morphology of the DPPy nanosheets. a) Schematic of the octadecylamine/PD nanosheet. b) PPy was assembled and polymerized on an octadecylamine/PD nanosheet. c,d) Three layers of polydopamine‐PPy nanosheet. e) SEM image of the nanosheet structures. Inset shows a membrane consisting of DPPy20 nanosheets. f) AFM topographic image of DPPy30 and the corresponding height profiles of the two nanostructures indicated with white lines.

We synthesized DPPy nanosheets using a bottom‐up self‐assembly approach (**Figure** **1**a–d). Octadecylamine and dopamine were preassembled into 2D bilayers at the water/ethanol interface using previously reported methods.^[^ ^11^ ^]^ The bilayer lamellae have a variety of exposed functional groups (catechol and amine), which interact with pyrrole monomers through hydrogen bonds and electrostatic force.^[^ ^12^ ^]^ The diffusion of pyrrole molecules onto the surface of the octadecylamine/PD substrate resulted in a 2D morphology. The polymerization of pyrrole was completed by adding FeCl_3_ and then incubating overnight at 4 °C. Scanning electron microscopy (SEM) micrographs of the synthesized DPPy suggested the formation of nanosheet morphology with narrow size distribution (Figure 1e). The resultant nanostructure displayed Janus properties with an asymmetric facial hydrophilicity and a black appearance due to visible light absorbance (Figure S1, Supporting Information). A free‐standing layered membrane was then prepared by vacuum filtration of a nanosheet suspension in the presence of a small amount of poly(vinyl alcohol) to show the flexibility of the resulting 2D nanosheets (inset in Figure 1e).^[^ ^13^ ^]^ Three types of lateral DPPy nanosheets with decreasing size distribution were prepared by adding 10, 20, and 30 µL pyrrole monomer in a 20 mL ethanol/water mixture denoted as DPPy10, DPPy20, and DPPy30, respectively. The light absorbance of the 2D nanostructures in the range up to 3000 nm increased as a larger amount of pyrrole was added (Figure S2, Supporting Information). SEM images validated a size decrease from 2.29 ± 1.70 to 0.68 ± 0.53 µm^2^ with an increasing amount of pyrrole monomer (Figure S3, Supporting Information). The atomic force microscopy (AFM) image further revealed a uniform lateral size of DPPy30 (Figure 1f). The AFM image of DPPy30 confirmed that the resultant 2D structures have a thickness of ≈3.8 nm (Figure S3, Supporting Information).

### 2.2 Optical Properties of DPPy Nanosheets

**Figure 2:**
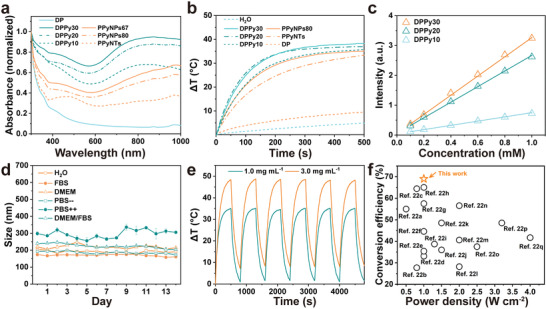
Characterization of DPPy nanosheets in solution. a) UV–vis‐NIR absorption spectra of various DPPy nanosheets compared with PPy nanospheres and nanotubes. b) Temperature increase of various materials in 2.0 mg mL^−1^ under 808 nm laser irradiation. c) The UV–vis absorption intensity of DPPy in water with increasing concentrations. Data shown as mean ± s.d., *N* = 3. d) Stability test as shown by size profiles of DPPy nanosheets in different buffer solutions recorded by dynamic light scattering measurements for 2 weeks. Data shown as mean ± s.d., *N* = 3. e) Repeated heating/cooling profiles of DPPy20 suspended in PBS with different concentrations under 808 nm laser irradiation with a power density of 0.5 W cm^−2^. f) Comparison of photothermal conversion efficiency under irradiation with various power densities of nanosheets reported in this work and in the past 5 years.^[^ ^22^ ^]^

The integration of PPy and PD was further demonstrated by FT‐IR spectra displaying the PPy characteristic peaks at 1079 cm^−1^ (C—H in plane vibration), 1465 cm^−1^ (vibration of the pyrrole ring), and 1542 cm^−1^ (C=C bond) (Figure S4, Supporting Information).^[^ ^14^ ^]^ The indole ring C—N—C stretching mode at around 1364 cm^−1^ and C=C stretching mode at 1456 cm^−1^ demonstrated the presence of PD.^[^ ^15^ ^]^ The slight shift of these peaks might be caused by the interaction between PPy and PD, suggesting that PPy had successfully been incorporated with the substrate.^[^ ^16^ ^]^ The characteristic absorption around 385 nm in the UV‐vis spectra could be attributed to the *π*–π* electronic transition of PPy.^[^ ^17^ ^]^ This donor–acceptor hybrid 2D structure enhanced the absorption of DPPy30 compared to that of tubular and spherical nanostructures, i.e., PPy nanoparticles with 67 nm (PPyNPs67) and 80 nm (PPyNPs80) diameter, and PPy nanotubes (PPyNTs) (Figure S5, Supporting Information). The enhanced absorption in the NIR region might be attributed to the reduced bandgap after the conjugation of PPy and PD (**Figure** **2**a).^[^ ^8^, ^18^ ^]^ The hybrid nanostructures might facilitate photo‐induced energy or electron transfer from PD to PPy, which has also been observed for graphene, oligothiophene, and porphyrin, etc.^[^ ^19^ ^]^ The strong light absorption of PD also contributed to the enhanced absorption of DPPy.^[^ ^20^ ^]^ Additionally, a higher ratio of pyrrole monomer could lead to higher light absorption in the NIR region (Figure 2a). The UV–vis–NIR spectra of various PPy nanostructures revealed a higher absorption efficiency of DPPy20 than PPy nanoparticles and nanotubes (Figure S6, Supporting Information).

This enhanced light absorption property and hybrid structure endowed the nanosheets with promoted photothermal conversion efficiency and stability. Under the irradiation of an 808 nm NIR laser (0.5 W cm^−2^), the temperature in the 2D nanostructure aqueous solution increased by 38.2 ± 0.06 °C from room temperature within 500 s, whereas the temperature of H_2_O increased by < 4 °C (Figure 2b). As the concentration of DPPy increased from 0.1 to 1.0 mm (calculated based on pyrrole monomers), the light absorption also increased linearly (Figure 2c; and Figure S6, Supporting Information) as did thetemperature change (Figure S7a–c, Supporting Information). Additionally, we compared the photothermal conversion efficiency under irradiation of 1064 nm with a power density of 0.3 W cm^−2^ to show the effectiveness of shapes on photothermal properties. Temperature changes of 35.4 ± 2.7 °C at 808 nm (0.3 W cm^−2^) and 28.2 ± 1.7 °C at 1064 nm (0.3 W cm^−2^) were recorded with 2.0 mg mL^−1^ of DPPy20, which was greater than tubular and spherical nanostructures under the same conditions (Figure S7d,e, Supporting Information). No apparent aggregation or salting‐out effect of DPPy was observed in various buffer solutions for 2 weeks (Figure 2d). The photothermal properties of DPPy were highly stable after six successive repeated cycling tests with exposure to an 808 nm laser (0.5 and 1.0 W cm^−2^) (Figure 2e). These results suggest that the stability of DPPy would satisfy the requirements of PTT.^[^ ^21^ ^]^ The photothermal conversion efficiency of DPPy20 at 808 nm was calculated to be 69%, which is also relatively high compared to other conductive polymer nanostructures. Specifically, we achieved a higher photothermal conversion efficiency than photothermal agents reported in the past 5 years (Figure 2f).

### 2.3 Enhancement of Photothermal Conversion and Therapeutic Performance

To investigate the potential of DPPy nanosheets as PTT agents, their biocompatibility and therapeutic efficacy were evaluated on 2D in vitro models. Negligible cell death after 24 and 48 h of incubation with HeLa and MEL‐246 cancer cell lines demonstrated that DPPy20 nanosheets with rising concentrations (0.02, 0.05, 0.1, 0.2, 0.5, 1.0, 2.0, and 5.0 mg mL^−1^) exhibited relatively high biocompatibility (Figure S8, Supporting Information). Further, a fluorescent LIVE/DEAD^M^ staining assay after incubation of MCF‐7 cells with 0.2 mg mL^−1^ DPPy20 for 24, 48, and 72 h revealed limited cell death (Figure S9, Supporting Information). No obvious difference in viability was found when HeLa cells were exposed to DPPy20 nanosheets at the concentration up to 0.2 mg mL^−1^ for 48 h (Figure S10, Supporting Information). We also found that all types of DPPy nanosheets (i.e., DPPy10, DPPy20, and DPPy30) showed low cytotoxicity after coincubation with MCF‐7 cells for 48 h at a concentration of 0.2 mg mL^−1^ (Figure S11, Supporting Information). The HeLa cells in the presence of laser irradiation without DPPy20 showed marginal changes in cell viability (Figure S12a, Supporting Information). In contrast, the cell viability decreased from ≈98% to 10% with irradiation of 808 nm (0.5 W cm^−2^) laser for 10 min after coincubation with DPPy20 for 24 h (Figure S12b, Supporting Information). These results were consistent with the fluorescent LIVE/DEAD^M^ staining assay, where severe HeLa cell death was found after incubation with DPPy20 followed by 808 nm laser irradiation at 0.5 W cm^−2^ (Figure S13, Supporting Information).

3D spheroid tumor models mimicking in vivo conditions were further employed to evaluate the PTT of the DPPy. Medium (245.2 ± 18.1 µm) and large (360.0 ± 32.0 µm) spheroids were generated by seeding 50 and 100 µL of HeLa, MCF‐7, or MEL‐246 cells (10^3^) suspension in ultralow attachment microplates (Figure S14, Supporting Information). No appreciable cell death was observed in cells exposed to the 808 nm laser alone or incubated with DPPy20 (Figure S14, Supporting Information). In contrast, spheroids treated with DPPy20 coupled with 808 nm laser irradiation (0.5 W cm^−2^) for 5, 10, 20, and 30 min exhibited substantial cell death (Figure S15, Supporting Information). Similarly, DPPy20 with a concentration of 0.2 mg mL^−1^ combined with 808 nm laser irradiation for 30 min at a power density of 0.5 W cm^−2^ exhibited photothermal cytotoxicity to MEL‐246 and MCF‐7 spheroids (Figure S16, Supporting Information). DPPy20 also showed a dose‐dependent photothermal effect for MCF‐7 spheroids. Increasing the concentration of DPPy20 from 0.01 to 0.2 mg mL^−1^ decreased the cell viability of MCF‐7 spheroids (Figure S17, Supporting Information). These results illustrate that the DPPy nanosheets are excellent candidates for PTT.

### 2.4 In Vivo Photoacoustic Imaging

**Figure 3:**
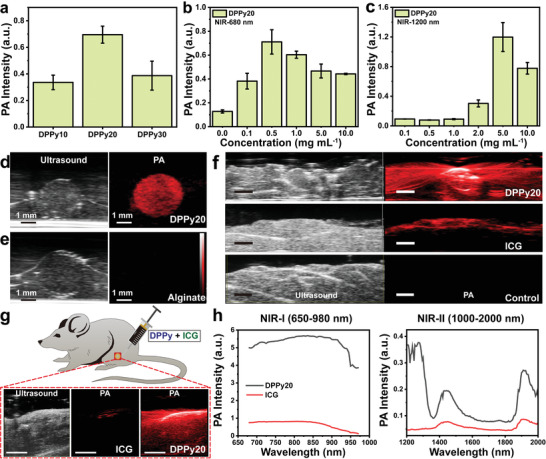
Performance of 2D nanosheets as contrast agents for PA imaging. a) PA intensity of DPPy10, DPPy20, and DPPy30. b,c) PA intensity of DPPy20 at wavelengths of 680 and 1200 nm. Data shown as mean ± s.d., *N* = 3. d) Representative ultrasound and PA image of DPPy20‐loaded alginate sphere phantom. e) Representative ultrasound and PA image of alginate sphere phantom not loaded with DPPy. f) In vitro ultrasound and PA image of chicken slices injected with 250 µL of 0.5 mg mL^−1^ aqueous solution of DPPy20 and ICG. Scale bar, 2 mm. A spectroscopic experiment was performed starting at 680 nm to a maximum of 2000 nm with a 5 nm step size. g) Representative in vivo PA images of a healthy mouse after intramuscular injection of a mixture of DPPy20 (1.0 mg mL^−1^) and indocyanine green (ICG) (1.0 mg mL^−1^) in the hind limb. Scale bar, 5 mm. h) Corresponding PA intensity in the first (650–980 nm, NIR‐I) and the second (1000‐2000 nm, NIR‐II) NIR window. Data were collected by injecting DPPy20 and ICG into 3 mice.

PA imaging is a promising modality for imaging biological structures, where the amplitude of the PA signal is proportional to the thermoelastic performance and absorbance capability of the contrast agent.^[^ ^23^ ^]^ The quick temperature rise resulting from the absorption of laser light by DPPy leads to thermoelastic expansion and ultrasonic emission, which can be detected by piezoelectric sensors and used to reconstruct a 3D image. Additionally, the broad optical absorption spectrum of our nanosheet structure extends to the secondary NIR window (NIR‐II), suggesting the nanosheets could be good contrast agents for NIR PA imaging both in the first (NIR‐I) and secondary NIR windows.^[^ ^24^ ^]^ We demonstrated that the PA intensity peaked at DPPy20 (**Figure** **3**a). A lower PA intensity for DPPy30 is likely due to a higher ratio of PPy in these nanosheets showing stronger NIR absorbance (Figure 2a). We measured the dose‐dependent PA signal of DPPy20 in alginate sphere phantoms at concentrations from 0.1 to 10.0 mg mL^−1^. Figure 3b,c shows the PA intensity of DPPy20 at 680 and 1200 nm, respectively. The highest intensity of PA signal was obtained with the phantom samples containing 0.5 mg mL^−1^ of DPPy20 at 680 nm (NIR‐I region) and 5.0 mg mL^−1^ of DPPy20 at 1200 nm (NIR‐II region) (Figure 3b,c). These observations might be attributed to DPPy nanosheets with a higher concentration absorbing more excitation light, which prevents PA signal from penetrating deeper within the sample, leading to lower signals. A comparison of their PA signal generation efficiency was demonstrated by the strong contrast intensity in alginate spheres loaded with DPPy20 (0.5 mg mL^−1^) and imaged at 680 nm (Figure 3d,e; and Movie S1, Supporting Information). As compared with PPy nanoparticles (PPyNPs67), the DPPy20 showed a higher PA intensity at the same concentration of 0.5 mg mL^−1^ (Figure S18, Supporting Information).

We injected chicken slices with 250 µL of DPPy20 in PBS at a concentration of 0.5 mg mL^−1^. PA images were acquired across a range of excitation wavelengths from 680 to 980 nm. As expected, a strong PA signal of DPPy20 was distinctly visualised in chicken breast tissue (Figure 3f). Slices with DPPy20 showed obvious contrast in injected areas compared with control slices injected with PBS or the commercially available PA contrast agent indocyanine green (ICG). PA signal could be detected at greater depth within the tissue following injection of DPPy20 versus ICG (Figure 3f; and Movie S2, Supporting Information).

To characterise the in vivo PA properties of DPPy, 20 µL of 1.0 mg mL^−1^ DPPy20 and 1.0 mg mL^−1^ ICG mixture was injected into a mouse hind limb. DPPy20 showed higher contrast in the muscle compared to ICG (Figure 3g). When DPPy20 and ICG were mixed and injected, spectral unmixing using the VevoLab software demonstrated that DPPy20 could be differentiated from ICG in the mouse hindlimb. DPPy20 nanosheets had stronger PA intensity in the NIR‐I window than ICG. Additionally, peaks around 1250 nm in the NIR‐II window verified that the PA imaging performance of DPPy20 outperforms that of the commercially available ICG (Figure 3h). This result demonstrated a potential use of the as‐fabricated DPPy as a PA contrast agent with lower optical scattering from biological substrates in the NIR‐II window and reduced tissue autofluorescence in the NIR‐I window.

### 2.5 Confocal Raman Visualization of DPPy Nanosheets

**Figure 4:**
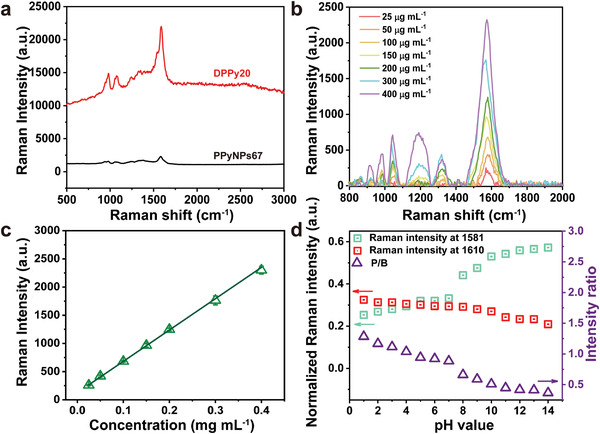
Raman shift and pH value relationship of DPPy20. a) Raman spectra of DPPy20 (red) and PPyNPs67 (black) using 532 nm laser and 5 s accumulation time. b) Relative Raman intensity of DPPy20 aqueous suspensions plotted as a function of the concentration of PPy. The Raman intensity data were collected using a 532 nm laser of 2 mW power with an integration time of 0.5 s three times, see the Supporting Information for more details about the measurement. c) Raman intensity of DPPy20 in PBS with increasing concentration of pyrrole. Data shown as mean ± s.d., *N* = 6. d) Relative Raman intensity ratio of vibrational bands of C=C at 1610 and 1581 cm^−1^, which could be assigned to the transition between polaron and bipolaron states.

In addition to PA imaging, our conductive polymer‐based 2D nanostructures also exhibited the potential to serve as Raman contrast agents which improves the spatial resolution of the images up to several micrometers. Remarkably, Raman spectroscopy is also a widely used tool to study conductive polymers associated with the presence of polarons and bipolarons.^[^ ^25^ ^]^ These structures provided detailed information about pH values and redox properties of the surroundings. DPPy20 increased Raman intensity under 532 nm laser excitation compared to PPyNPs67 (**Figure** **4**a). The peak at 1580 cm^−1^ assigned to the typical C=C backbone of PPy shows a Raman enhancement of nearly two orders of magnitude. The Raman scattering enhancement of the DPPy20 monolayers might be explained by the electron transfer between the two layers.

The Raman intensity of DPPy can be regulated both by the concentrations and pH values of the surroundings. A linear relationship was found between Raman intensity and DPPy20 concentrations between 0.025 and 0.4 mg mL^−1^, which indicated good dispersity of DPPy in aqueous solutions (Figure 4b,c). To remove any instrument effects, we used the intensity ratio of vibrational bands of C=C at 1610 and 1581 cm^−1^ (polarons and bipolarons, respectively).^[^ ^25b^ ^]^ As shown in Figure 4d (Figures S19 and S20, Supporting Information), the ratio (P/B) slightly decreased as the pH values increased, reflecting the change of the protonation/deprotonation units in the resultant nanosheet structures. In lower pH environments, PPy was protonated as evidenced by the C=C band shifting from 1610 to 1581 cm^−1^.^[^ ^26^ ^]^ Raman spectra of DPPy20 excited with a 532 nm laser reflected the consecutive deprotonation of PPy with increasing pH values as two new peaks appeared at 1548 and 1410 cm^−1^, corresponding to the increasing number of C=N bonds in the neutral units.^[^ ^27^ ^]^ In an alkaline environment, the potential for PPy protonation was significantly reduced, as reflected by the C—C band shifting to 1488 cm^−1^.^[^ ^28^ ^]^ In strong alkaline dispersion with a pH value approaching 14, a broad fluorescence band appeared and the original bands of PPy nearly disappeared due to the excitation of the overoxidized units.^[^ ^29^ ^]^ The double peaks at 1052 and 1083 cm^−1^ were assigned to the C—H in‐plane deformation, and another double peak at 1330 and 1370 cm^−1^ were attributed to the ring‐stretching mode of PPy.^[^ ^30^ ^]^

**Figure 5:**
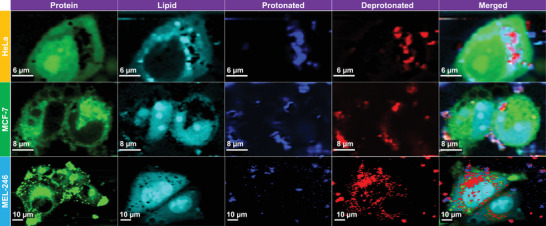
Confocal Raman visualization of HeLa, MCF‐7, and MEL‐246 cells treated with 0.1 mg mL^−1^ of DPPy20. Protein, lipid, protonated DPPy20, and deprotonated DPPy20 were selected to display. Protein‐rich regions at 1006 and 679 cm^−1^ (shown in green), lipid‐rich regions at 1300 and 1430 cm^−1^ (shown in cyan), intensity ratio of vibrational bands of C=C at 1610 and 1581 cm^−1^ higher than 0.88 for protonated DPPy20 regions (shown in blue), while lower than 0.88 for deprotonated DPPy20 regions (shown in red). See the Supporting Information for more details. The Raman intensity of each component is normalized to the lipid peak intensity.

The typical Raman spectra of DPPy nanosheets could thus provide information about pH values in the cellular microenvironment. We examined components of HeLa, MCF‐7, and MEL246 cells by confocal Raman microscopy (**Figure** **5**). The strong Raman shift of PPy in DPPy20 could be used to confirm the presence of the nanosheets. Protonated and deprotonated PPy were observed inside and outside the cells (Figure S21, Supporting Information), which indicates an acidic extracellular environment and mildly alkaline intracellular environment around HeLa cells, consistent with previous reports.^[^ ^31^ ^]^ We then performed 3D Raman imaging of HeLa cells using DPPy20 as the probe (Movie S3–S6, Supporting Information). The intensities of specific Raman peaks were reconstructed to highlight the main components of cells and visualize the 3D geometry. PA imaging can monitor tissues at several centimeters of depth but is limited in spatial resolution. Raman imaging could offer higher spatial resolution and more detailed tumor microstructures.

## 3 Conclusion

In summary, we reported a self‐assembled hybrid 2D nanosheet consisting of PD and PPy (DPPy). Our DPPy nanosheet has a higher photothermal conversion efficiency and light absorbance than tubular and spherical structures. The improved visible and NIR light absorbance of our DPPy nanosheet resulted in reduced bandgap energy and thus enhanced photothermal conversion and phototherapy efficacy. In vitro and in vivo experiments confirmed that DPPy nanosheets improve PA intensity when injected into tissues compared to a commercially available imaging contrast agent (ICG). Furthermore, the tunable polaron and bipolaron band of PPy provided a strategy to probe the pH values in cell surroundings using confocal Raman spectroscopy due to their sensitivity to pH environments. The development of this conductive nanostructure with intrinsic PA imaging and Raman imaging properties will potentially warrant dual image‐guided efficient PTT of cancer.

## 4 Experimental Section

### Materials

Octadecylamine, dopamine hydrochloride, NaOH, ammonium persulfate (APS, 98%), pyrrole, iron (III) chloride hexahydrate, methyl orange sodium 4‐[(4‐dimethylamino)phynylazo] benzenesulfonate (MO), indocyanine green (ICG), and polyvinyl alcohol (PVA, Mw, 2400, 13 000, and 31 000 g moL^−1^) were purchased from Sigma‐Aldrich and used without any pretreatment. All chemical reagents used in this paper are of analytical purity. Pierce methanol‐free 16% formaldehyde w/v (PFA), Gibco Dulbecco's Modified Eagle Medium (DMEM), Dulbecco's phosphate buffered saline (DPBS) without phenol red, calcium, and magnesium (Gibco), Dulbecco's phosphate buffered saline (DPBS) with calcium and magnesium (Gibco), GlutaMAX (Gibco, 31966‐021), LIVE/DEAD viability cytotoxicity assay kit including calcein‐AM and Ethidium homodimer‐1 (EthD‐1), and AlamarBlue dye were purchased from Thermo Fisher Scientific.

### Preparation of Polydopamine‐Polypyrrole Nanosheets

Typically, 20 mg of octadecylamine was dissolved in 4 mL of ethanol. The solution was added to 16 mL H_2_O. The self‐assembly of octadecylamine nanosheets was allowed at room temperature for 30 min. 24.5 mg of dopamine hydrochloride was added to the above solution, followed by stirring at room temperature for another 30 min. After stirring, 0.3 mL of NaOH (1.0 m) was added, and the above solution eventually turned dark after continuous stirring at room temperature for 24 h, indicating the polymerization of dopamine into polydopamine. Subsequently, the pH value of the mixture was tuned to 4.0 using HCl (1.0 m), into which 30 µL pyrrole monomer and 16.8 mg of ammonium persulfate (or 31 mg of FeCl_3_∙H_2_O) were added. The polymerization of pyrrole was completed in a fridge (4 °C) overnight. Final samples were separated by high‐speed centrifugation at 14 000 rpm for 20 min and repeatedly washed with ethanol and water three times, respectively. The resulting nanosheets were named DPPy30. Nanosheets prepared by feeding the reaction with 10 and 20 µL pyrrole monomers were named DPPy10 and DPPy20, respectively. Thicker PPy nanosheets with a thickness of about 40 nm were obtained by following the same procedure without adding octadecylamine.

### Preparation of PVA/DPPy Membrane

PVA with molecular weight of 2400 g moL^−1^ was dissolved in water at 90 °C to give a 5 wt% solution. A uniform mixture of DPPy and PVA was prepared by adding 5 mL of PVA solution to 5 mL of DPPy suspension (1.0 mg mL^−1^) and subjected to sonication for about 30 min at room temperature. A free‐standing PVA/DPPy composite membrane was produced using vacuum filtration of the suspension. The obtained membrane was dried overnight under a vacuum without further hot press.

### Preparation of PPy Nanotubes

The reference PPyNTs were prepared according to a previous report.^[^ ^32^ ^]^ Briefly, 50 mL of an aqueous solution containing 0.05 m iron (III) chloride and 0.0025 m MO (0.082 g) was prepared. 0.34 mL of pyrrole monomer was added dropwise into the solution under stirring. The mixture was subjected to an ultrasonic bath for 5 min and shaken by hand to dissolve all the reagents. The process of polymerization was carried out at room temperature for 24 h. The products were then filtered and washed with 0.2 m hydrochloric acid until no MO could be detected by UV–vis spectroscopy.

### Preparation of PPy Nanoparticles

Uniform PPy nanoparticles were synthesized via a PVA stabilized method.^[^ ^33^ ^]^ Briefly, 0.75 g PVA was dissolved in 20 mL H_2_O at 60 ^°^C overnight, and then cooled to room temperature. PPyNPs80 were prepared using PVA with a molecular weight of 89 000–98 000. PPyNPs67 were prepared using PVA with a molecular weight of 146 000–186 000 g moL^−1^. 0.63 g FeCl_3_∙H_2_O was then added, followed by stirring for 30 min at room temperature and cooling down to 4 ^°^C. 60 µL pyrrole was added dropwise to the solution. The polymerization proceeded at 4 ^°^C overnight. The resulting nanoparticles were separated by centrifugation (15 000 rpm, 40 min); washed with hot water several times; and resuspended in water by ultrasonication for 3 min.

### Water Contact Angle Measurement

The wettability of the Janus polypyrrole‐polydopamine membrane was investigated by water contact angle measurement. The water contact angles of the dried nanosheets after spin‐coating with aqueous and toluene dispersions on the surface of the silicon wafer were measured at room temperature using a water contact angle measuring instrument. A water drop with a volume of 10 µL was placed onto the surface with a microsyringe in the air. The measurement was repeated at least three times for each surface ≈10 s after the water droplets contacted the surface.

### Photothermal Effect of DPPy in Solution

The photothermal efficiency of DPPy with different ratios of polypyrrole (i.e., DPPy10, DPPy20, and DPPy30) at different concentrations (0.0, 0.05, 0.1, 0.2, 0.5, 1.0, 2.0, and 3.0 mg mL^−1^) were initially confirmed by a probe thermometer (Omega, HH303), where the output energy of the 808 and 850 nm was set as 0.5 W cm^−2^ with different irradiation time, respectively. The photothermal stability of DPPy20 nanosheets (1.0 and 3.0 mg mL^−1^) was confirmed by cycle irradiation with an 808 nm laser.

### Photothermal Conversion Efficiency Studies

The photothermal conversion efficiency was determined according to a previous report.^[^ ^4^ ^]^ 200 µL H_2_O containing 1.0 mg mL^−1^ of distinct DPPy (DPPy10, DPPy20, and DPPy30) was exposed to laser irradiation at 808 nm (0.5 and 1.0 W cm^−2^). The temperature was recorded using the thermometer (Omega, HH303) every 10 s until reaching the maxima. The photothermal conversion efficiencies were calculated as

(1) $$\eta =\frac{hS\left(T_{\max}-T_{\mathrm{surr}}\right)}{I\left(1-10^{\left(-A_{808}\right)}\right)}$$

Where *η* is the photothermal conversion efficiency; *h* is the heat transfer coefficiency (glass vial with thickness of 1 mm was used in the work, 6.6 W m^−2^ K^−1^); *S* is the surface area of the container (11.6 mm in diameter, and 32 mm in height for this work); *T*_max_ is the maximum steady state temperature of the dispersions; *T*_surr_ is the ambient temperature (25 °C); *I* is the laser power used for the experiment; and *A*_808_ is the absorbance at 808 nm.

### In Vitro Cytotoxicity Assay

The cytotoxicity of the DPPy nanosheets in vitro was investigated using an AlamarBlue assay. Hela, MCF‐7, and MEL246 cells were maintained in MDEM (Gibco) supplemented with 10% v/v FBS and penicillin/streptomycin (P/S, Gibco). HeLa cells (1000 per well), MCF‐7 cells (1000 per well), and MEL‐246 cells (1000 per well) were incubated in a 96‐well plate overnight. The old medium was changed with the medium containing DPPy and fresh medium. Cells cultured in complete medium without DPPy were used as a blank control. The concentrations of the DPPy20 nanosheets were set as 0.0, 0.02, 0.05, 0.1, 0.2, 0.5, 1.0, 2.0, and 5.0 mg mL^−1^. After exposure for another 24 and 48 h at 37 °C, the supernatant was removed and then carefully rinsed with PBS 3 times. Then, complete medium (90 µL) and AlamarBlue reagent (10 µL) were added. After incubation for another 2 h, the cell viability was analyzed by measuring the fluorescence intensity at 590 nm. The relative cell viability was expressed as

(2) $$\mathrm{Cell}\;\mathrm{viability}\;\left(\%\right)=\frac{FL_{\exp}-FL_{\mathrm{Rea}}}{FL_{\mathrm{con}}-FL_{\mathrm{Rea}}}\times 100\%$$

Where *FL*_exp_ is the fluoresence intensity of the experiment sample; *FL*_con_ is the fluorescence intensity of the control group; and *FL*_Rea_ is the fluoresence intensity of complete medium (90 µL) plus AlamarBlue reagent (10 µL). The average value was obtained from 6 parallel samples.

The cytotoxicity of DPPy20 with extended incubation time was further investigated using Calcein‐AM/EthD‐1 labeling method. Briefly, MCF‐7 cells (1 × 10^5^ cells cm^−2^) were seeded into 6‐well plates and allowed to settle overnight. DPPy20 with a concentration of 0.2 mg mL in complete medium were added into the plates. After exposure for another 24, 48, and 72 h, the cells were stained by calcein‐AM (live cells) and EthD‐1 (dead cells) according to the manufacturer's suggested procedures. The samples were imaged using a Zeiss LSM700 fluorescence microscope. The cytotoxicity of DPPy20 with increasing concentrations (0.02, 0.05, 0.1, and 0.2 mg mL^−1^) to HeLa cells was further confirmed using Calcein‐AM/EthD‐1 labeling method. HeLa cells were seeded into 6‐well plate (1 × 10^5^ cells cm^−2^) and were allowed to adhere overnight. Then, the original medium was removed and culture medium containing DPPy20 with increasing concentration was added into each well. Cells were further exposed to DPPy20 for 48 h and were stained with Calcein‐AM and EthD‐1. The cytotoxicity of DPPy10, DPPy20, and DPPy30 to MCF‐7 cells (1 × 10^5^ cells cm^−2^) was studied following the same procedure.

### In Vitro Photothermal Killing Efficiency Studies

The photothermal killing efficiency for both cell monolayers and spheroids was assessed. In cases of cell monolayers, HeLa cells were cultured on a 96‐well plate at a density of 1 × 10^4^ cells per well with 5% CO_2_ at 37 °C for 24 h. The cells were washed with warm PBS before the addition of 100 µL of DPPy20 in complete culture medium at a concentration of 0.02, 0.05, 0.1, 0.2, 0.5, 1.0, 2.0, and 5.0 mg mL^−1^. After 24 h incubation at 37 °C, the culture medium was removed and cells were washed with warm PBS several times. Each well was then filled with 100 µL culture medium and subjected to 808 nm laser irradiation at 0.5 W cm^−2^ for 10 min. After incubation overnight, cell viability was measured using AlarmaBlue assay and Live/Dead staining as described above.

To mimic in vivo conditions, 3D spheroids (HeLa, MCF‐7, and MEL246) were generated according to a previous report with slight modifications.^[^ ^34^ ^]^ Briefly, 50 and 100 µL of complete medium containing 1.0 × 10^3^ cells were seeded in an ultralow attachment 96‐well plate (Corning 96‐well Clear Round Bottom Ultra‐Low Attachment Microplate, NY) to obtain medium and large spheroids. Cells were spun down at 300 × g for 3 min and cultured with 5% CO_2_ at 37 °C for 24 h. 50 µL of fresh complete medium was added into each well after 24 h. The cytotoxicity of DPPy20 and effect of 808 nm laser irradiation individually were studied in these spheroids before the photothermal killing efficiency was investigated. Briefly, spheroids were collected, washed with PBS, and irradiated at 808 nm (0.5 W cm^−2^) for 5, 10, 20, and 30 min. Cell damage caused by DPPy20 was determined by culturing cells with nanosheets at 5% CO_2_ and 37 °C for 24 h. Cell viability was determined by labeling the cells with the LIVE/DEAD kit. Spheroids were observed under a confocal microscope (Confocal Zeiss LSM 700, Zeiss, Germany). Spheroids that were neither irradiated or treated with DPPy20 were also imaged (referred as the control). To determine the photothermal effect, the spheroids were cultured in complete medium containing DPPy20 (0.01, 0.02, 0.1, and 0.2 mg mL^−1^). After 24 h of incubation, spheroids were collected, washed with warm PBS, and treated by laser irradiation (808 nm, 0.5 W cm^−2^) for another 30 min. Cell damage produced by photothermal treatment was then determined under a confocal microscope according to the above procedure.

### In Vitro Photoacoustic Imaging

PA images were acquired at the UCL Centre for Advanced Biomedical Imaging using a high‐frequency ultrasound and nano‐second pulsed PA laser system (Vevo 3100, VevoLAZR, FujiFilm VisualSonics Inc., Toronto). To test the PA properties of DPPy, a range of DPPy formulations and concentrations were mixed with 2% alginate solutions (Sigma) and crosslinked in 100 mm CaCl_2_ to generate alginate sphere phantoms. A spectroscopic experiment was performed starting at 680 nm to a maximum of 2000 nm with a 5 nm step size (Probe: MX550S, Frequency: 40 MHz, Depth: 9 mm, Acquisition mode: Spectro). To determine whether DPPy could be detected at depth in biological tissue samples, 250 µL of 0.5 mg mL^−1^ aqueous solution of DPPy20 and ICG were injected into food grade chicken breast slices purchased from a butcher. Pure chicken breast was used as a reference. Three repeats were obtained for each sample. (Probe: MX250S, Frequency: 21 MHz, Depth: 13 mm, Acquisition mode: Spectro).

### In Vivo Photoacoustic Imaging

All animal studies were approved by the University College London Biological Services Ethical Review Committee and licensed under UK Home Office regulations and the Guidance for the Operation of Animals (Scientific Procedures) Act 1986 (Home Office, project licence No. PP692884, London). Healthy, 14‐week‐old female BALB/c mice were anaesthetised with 2% isoflurane in O_2_, hair was removed from the hindlimbs and mice were positioned supine on a heated imaging platform where temperature, respiration and heart rate were monitored. In 3 mice, 20 µL of DPPy20 (1.0 mg mL^−1^) was injected intramuscularly into the left hindlimb using a 29‐gauge needle. 20 µL of ICG (1.0 mg mL^−1^) was delivered in the same manner into the right hindlimb. PA imaging was performed before and immediately after injection using a FujiFilm Visualsonics Vevo 3100 high‐resolution ultrasound with a LAZR‐X photoacoustic imaging system at excitation wavelengths between 680 and 2000 nm (Probe: MX550S, Frequency: 40 MHz, Depth: 9 mm, Acquisition mode: Spectro). To investigate the PA imaging difference of DPPy and ICG, a mix of 20 µL DPBS containing 1.0 mg mL^−1^ DPPy20 and 20 µL 1.0 mg mL^−1^ ICG was injected intramuscularly into one additional mouse and imaging was repeated 3 times.

### 4.1 Confocal Raman Imaging

Raman spectra were recorded on a confocal Raman micro‐spectroscope (alpha300R+, WITec, Ulm, Germany) according to the previous report.^[^ ^9a^ ^]^ This system used 532 and 785 nm light source with a × 63/1.0 NA water immersion microscope objective lens (W Plan‐Apochromat, Zeiss, Oberkochen, Germany) and 40 mW laser power at the sample. The pinhole consists of a 50 µm fiber providing confocality to direct scattered light to the spectrometer. Both images and spectra were acquired using a thermoelectrically cooled back‐illuminated CCD camera (Newton DU970N‐BV‐353, Andor, Belfast, UK) with a spectral resolution of ≈10 cm^−1^. Cells were imaged by collecting Raman images from 5 layers of 1 µm increments in the *z*‐direction, thus spanning the cell volume. A 650 nm step size was used in the *x* and *y* direction for each Raman image with 0.3 s integration time and a spectral range from 0 to 3000 cm^−1^. Concentration‐dependent Raman intensity measurement was carried out using a Raman spectrometer system purchased from Ocean Insight. The device was composed of a 532 nm laser (190 µm in diameter and 2.2 mm in a depth of field) with tunable output power, Raman coupled fiber probe, Preconfigured QEPRO for 532 nm Raman, and a sample holder (9.5 mm in diameter). The Raman intensity data were collected using a 532 nm laser of 2 mW power with an integration time of 0.5 s three times.

### Raman Data Analysis

All Raman images were preprocessed by baseline correction, removal of cosmic rays (manually), and smoothing. Regarding the baseline correction, a third order polynominal processing method was used in the range of 700–1800 cm^−1^, which is a weighted least squares processing method set in the confocal Raman software. The smoothing was committed using a second order Savitzky–Golay algorithm with a 3‐point window, which is predefined in the WITec (Ulm, Germany) software. The spectra dataset was normalized to the peak of lipid to remove any instrument effects and ensure the comparability of the intensity. The typical phenylalanine and amide bands with strong CH_2_ twists and deformation at around 1300 and 1430 cm^−1^ were used to identify cellular structures, and the additional shoulder at 1130 cm^−1^ from cholesteryl stearate was used to resolve lipid subtypes. The highly specific molecular markers of phenylalanine (1006 and 679 cm^−1^) were used to show the presence of protein. The intensity ratio of vibrational bands of C=C at 1610 and 1581 cm^−1^, which could be used to describe the doping degree of DPPy nanosheets.^[^ ^25b^ ^]^

### Characterization

UV–vis spectra were recorded with a SpectraMax M5 (Molecular Devices) in the range of 300 and 1000 nm. Dynamic light scattering was performed using a ZetaSizer Nano ZS (Malvern Instruments Ltd.) to study the radius of the nanosheets and stability in various dispersions. Images of the nanosheets were obtained using a JSM6010LA scanning electron microscopy (JEOL). Attenuated total reflection Fourier‐transform infrared spectra of the nanosheets were collected on a Perkin Elmer Spectrum 100 FT‐IR spectrometer equipped with a diamond crystal insert. Atomic force microscopy (Agilent 5500) was used to investigate the thickness of the resulted nanosheets. Images were taken at 10, 5, and 2 µm. The UV–vis–NIR spectra were obtained by 508 PV microscope spectrophotometer (CRAIC Technologies).

### Statistical Analysis

Statistical analyses were carried out using ANOVA after proof of homogeneity of variances and normality tests using Origin. All the data in the study were presented as the mean ± standard deviation from at least three independent replicates (*n* ≥ 3). For all the other experiments, if not otherwise indicated, Student's paired or unpaired *t*‐test was used to evaluate individual differences between the means, and *p* < 0.05 was considered significant.

## Conflict of Interest

The authors declare no conflict of interest.

## Supporting information

Supporting Information

Supplemental Movie 1

Supplemental Movie 2

Supplemental Movie 3

Supplemental Movie 4

Supplemental Movie 5

Supplemental Movie 6

## Data Availability

The data that support the findings of this study are available from the corresponding author upon reasonable request.
