# Supplementary material for: Hybrid Polypyrrole and Polydopamine Nanosheets for Precise Raman/Photoacoustic Imaging and Photothermal Therapy
Source: Adv Healthc Mater. 2023 Jun 20;12(27):2301148. doi: 10.1002/adhm.202301148 (PMC11468501; doi:10.1002/adhm.202301148)
Supplement: Supplementary file 1 — Supporting Information [file ADHM-12-2301148-s003.pdf]

ADVANCED HEALTHCARE MATERIALS

Supporting Information

for *Adv. Healthcare Mater.*, DOI 10.1002/adhm.202301148

Hybrid Polypyrrole and Polydopamine Nanosheets for Precise Raman/Photoacoustic Imaging and Photothermal Therapy

*Hongya Geng, Emily J. Lupton, Yun Ma, Rujie Sun, Christopher L. Grigsby, Giulia Brachi, Xiaorui Li, Kun Zhou, Daniel J. Stuckey and Molly M. Stevens**

Supporting Information
©Wiley-VCH 2019
69451 Weinheim, Germany

Hybrid polypyrrole and polydopamine nanosheets for precise Raman/photoacoustic imaging and photothermal therapy

Hongya Geng, Emily Lupton, Yun Ma, Rujie Sun, Christopher L. Grigsby, Giulia Brachi, Xiaorui Li, Kun Zhou, Daniel Stuckey, and Molly M. Stevens*

Abstract: The development of near-infrared light (NIR)-responsive conductive polymers provides a useful theranostic platform for malignant tumours by maximizing spatial resolution with deep tissue penetration for diagnosis and photothermal therapy. Herein, we demonstrated the self-assembly of ultrathin two-dimensional (2D) polypyrrole nanosheets utilizing dopamine as a capping agent and a monolayer of octadecylamine as a template. The 2D polypyrrole-polydopamine nanostructure (DPPy) had tunable size distribution which showed strong absorption in the first and second near-infrared windows, enabling photoacoustic imaging and photothermal therapy. The hybrid double-layer was demonstrated to increase Raman intensity for 3D Raman imaging (up to two orders of magnitude enhancement and spatial resolution up to 1 µm). The acidic environment drove reversible doping of polypyrrole, which could be detected by Raman spectroscopy. The combined properties of the nanosheets could substantially enhance performance in dual-mode Raman and photoacoustic guided photothermal therapy, as shown by the 69% light to heat conversion efficiency and higher cytotoxicity against cancer spheroids. These pH-responsive features highlight the potential of 2D conductive polymers for applications in accurate, highly efficient theranostics.

DOI: 10.1002/anie.2016XXXXX

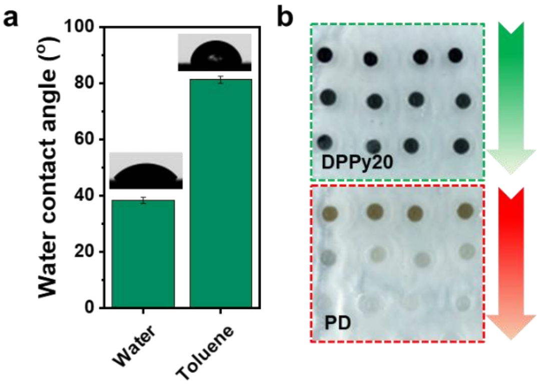

Figure S1. a, Water contact angle of the resultant nanosheet membrane coated on a silicon wafer prepared by re-suspending the material in water and toluene. Data shown as mean ± S.D. n = 3. b, Optical images of DPPy20 and polydopamine (PD) alginate beads with a concentration of 1.0 mg mL^{-1}, 0.5 mg mL$^{-1,}$ and 0.1 mg mL^{-1} from the top to the bottom.

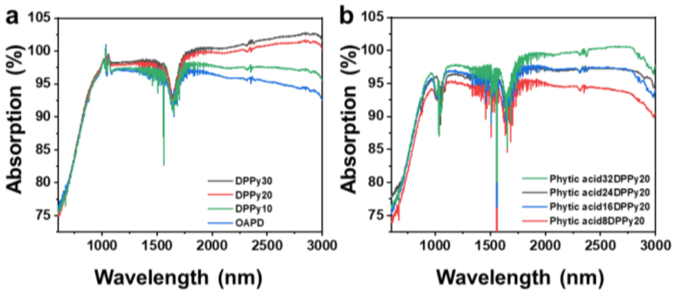

Figure S2. a, Absorption spectra of DPPy10, DPPy20, and DPPy30 . b, Absorption of DPPy20 doped by different amounts of phytic acid for 3 h at room temperature, i.e., Pyhtic acid 8DPPy20 (8 µL phytic acid + 20 µL pyrrole), Pyhtic acid16DPPy20 (16 µL phytic acid + 20 µL pyrrole), Pyhtic acid24DPPy20 (24 µL phytic acid + 20 µL pyrrole), and Pyhtic acid32DPPy20 (32 µL phytic acid + 20 µL pyrrole).

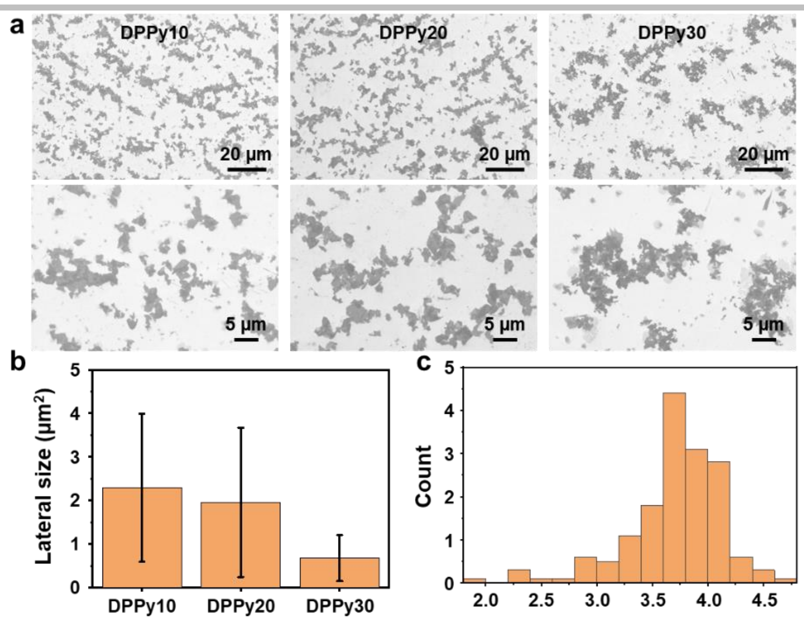

Figure S3. a, SEM images of DPPy10, DPPy20, and DPPy30. b, Lateral sizes of the as-prepared nanosheets measured by ImageJ. Data shown as mean ± s.d., n = 40. c, Height profile of DPPy20 nanosheets measured by AFM. N = 160 nanosheets measured.

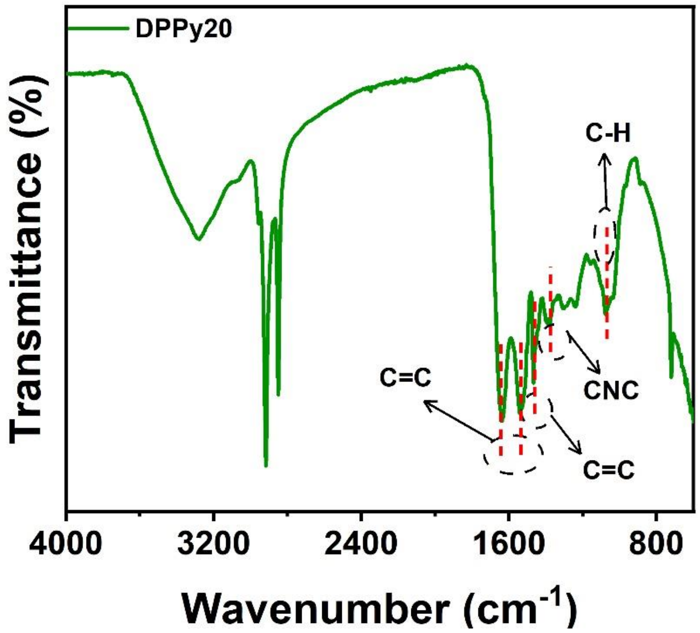

Figure S4. Fourier transform infrared spectroscopy (FTIR) spectra of DPPy20.[1]

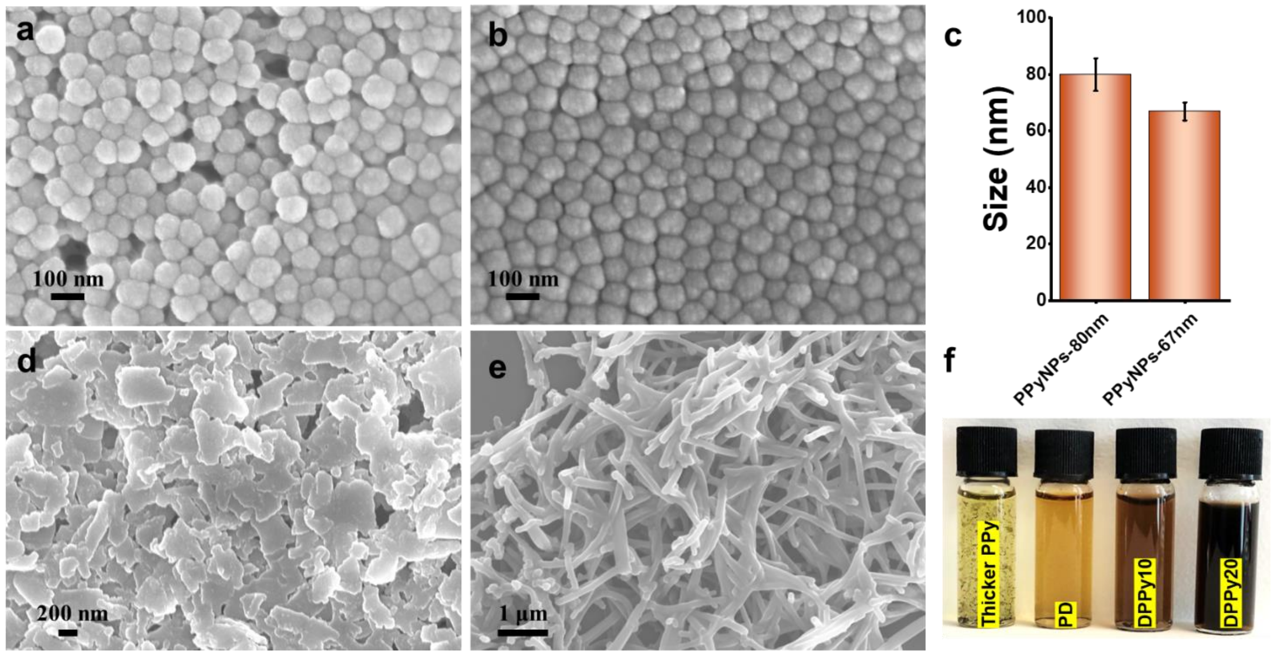

Figure S5. SEM images of as-prepared PPy nanostructure. a, PPy spheres with 79 ± 6.1 nm in diameter (PPyNPs80). b, PPy spheres with 67.0 ± 3.2 nm in diameter (PPyNPs67). c, Average of size of PPyNPs80 and PPyNPs67. Data shown as mean ± s.d., n = 15. d, Typical SEM image of thicker PPy nanosheets. e, Typical SEM image of PPy nanotubes. d, A typical SEM image of PPy thicker layer prepared without the addition of octadecylamine as the tempate. e, A typical SEM image of PPy nanotubes. f, PPy suspensions with a concentration of 0.1 mg mL^{-1}.

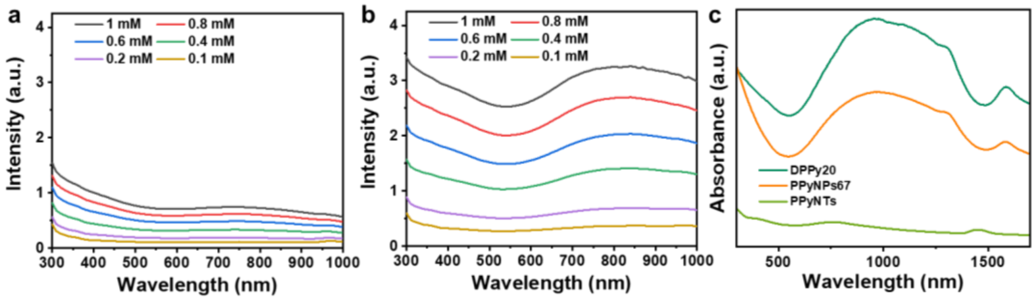

Figure S6. The UV-vis-NIR spectrum of various DPPy10 (a) and DPPy30 (b) dispersions with increasing concentrations. The UV-vis absorption spectra show the average of 3 repeats. c, Comparison of Vis-NIR absorption spectrum of DPPy20, PPyNPs67, and PPyNTs.

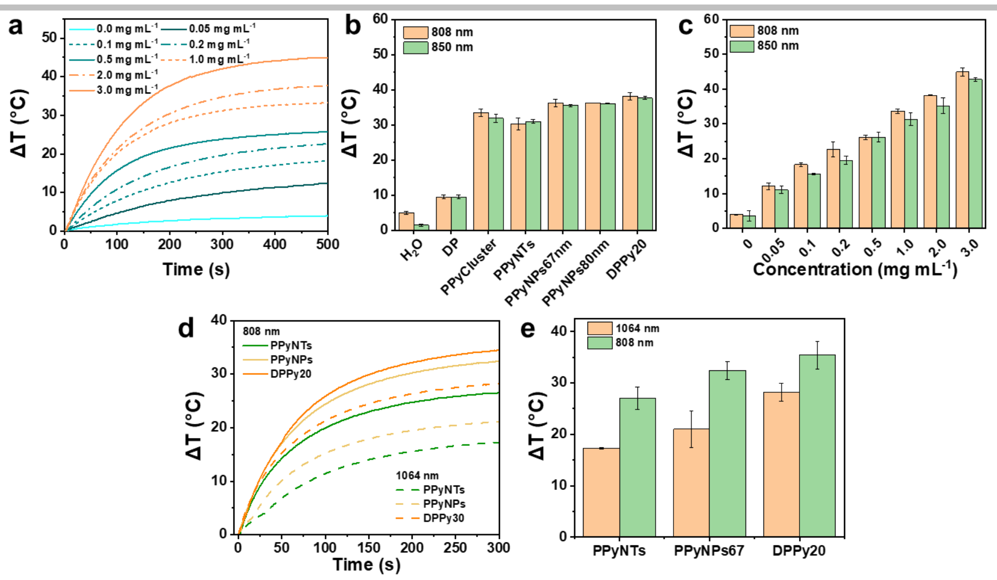

Figure S7. a, Photothermal heating effect of DPPy20 at a different concentration under 808 nm laser (0.5 W cm^{-2}). Data shown as mean of 3 measurements. b, Temperature changes of water dispersions of various samples (2.0 mg mL^{-1}) measured by irradiating suspensions under 808 and 850 nm laser for 500 s. c, Temperature changes of the DPPy20 water dispersions with increasing concentrations measured by irradiating for 500 s. Data shown as mean ± s.d., N = 3 independent experiments. d, Comparison of photothermal performances under 808 nm (0.3 W cm^{-2}) and 1064 nm (0.3 W cm^{-2}) laser irradiation. e, Temperature changes of various samples in figure d. Data shown as mean ± s.d. n = 3.

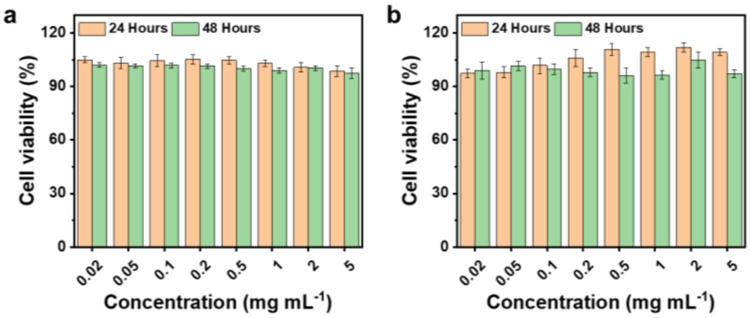

Figure S8. a, Cell cytotoxicity of HeLa cells in medium containing DPPy20 with increasing concentrations. b, Cell cytotoxicity of MEL246 cells in medium containing DPPy20 with increasing concentrations. The cell viability was measured using AlamarBlue assay. Data shown as mean ± s.d., N = 6 independent experiments.

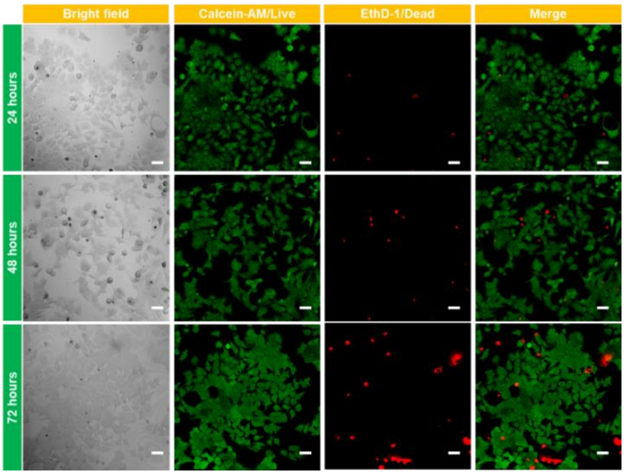

Figure S9. Confocal laser scanning microscopy (CLSM) images of MCF-7 cells (1×10^5 cells cm^{-2}) after incubation with DPPy20 (0.2 mg mL^{-1}) for different periods. Cell were stained with calcein-AM/EthD-1. Scale bar, 50 µm.

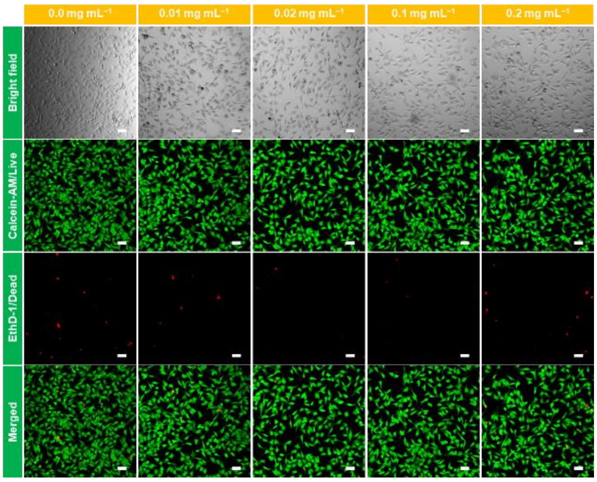

Figure S10. CLSM images of HeLa (1×10^5 cells cm^{-2}) being co-cultured in media containing increasing concentration of DPPy20 for 48 h. Cell were stained with calcein-AM/EthD-1. Scale bar, 50 µm.

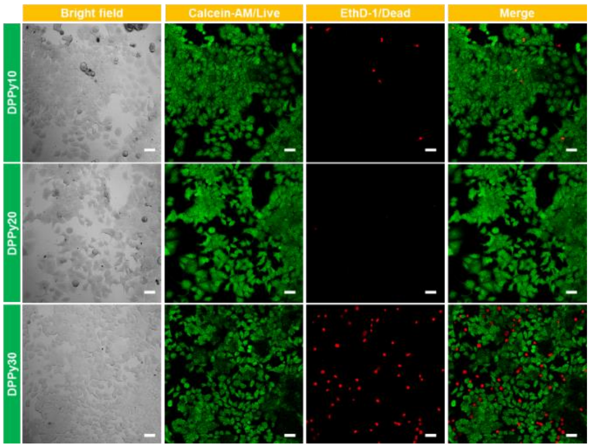

Figure S11. CLSM images of MCF-7 cells (1×10^5 cells cm^{-2}) incubated with DPPy10 (0.2 mg mL^{-1}), DPPy20 (0.2 mg mL^{-1}), and DPPy30 (0.2 mg mL^{-1}) for 48 h. Cells were stained with calcein-AM/EthD-1. Scale bar, 50 µm.

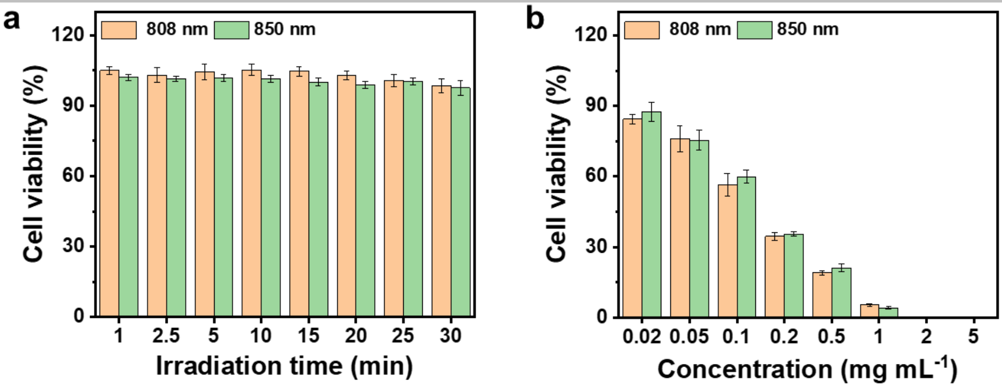

Figure S12. Photothermal therapy efficiency test of DPPy20. a, Relative viabilities of HeLa cells after being irradiated with 808 nm and 850 nm laser for the different times. b, Relative cell viability of HeLa cells incubated with DPPy20 at increasing concentrations with 808 nm and 850 nm laser irradiation at a power density of 0.5 W cm^{-2} for 10 min. Data shown as mean ± s.d., N = 6 independent experiments.

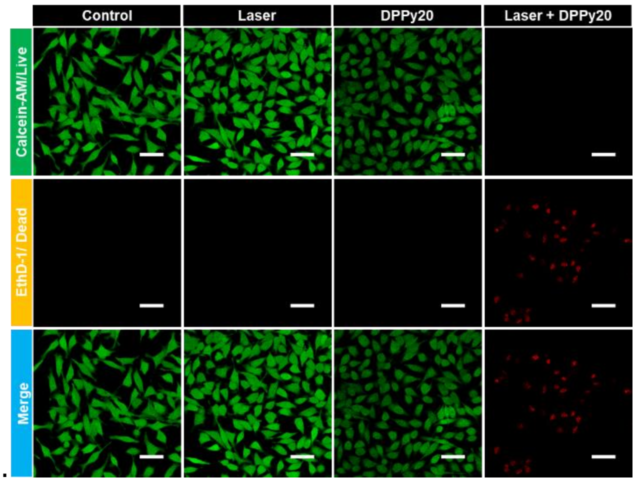

Figure S13. CLSM images of calcein-AM/EthD-1 stained HeLa cells. HeLa cells incubated with DPPy20 at 0.2 mg mL^{-1} with/without 808 nm laser irradiation at 0.5 W cm^{-2} for 10 min. HeLa cells in complete culture medium without DPPy20 and laser irradiation were used as control. Scale bar is 50 μm.

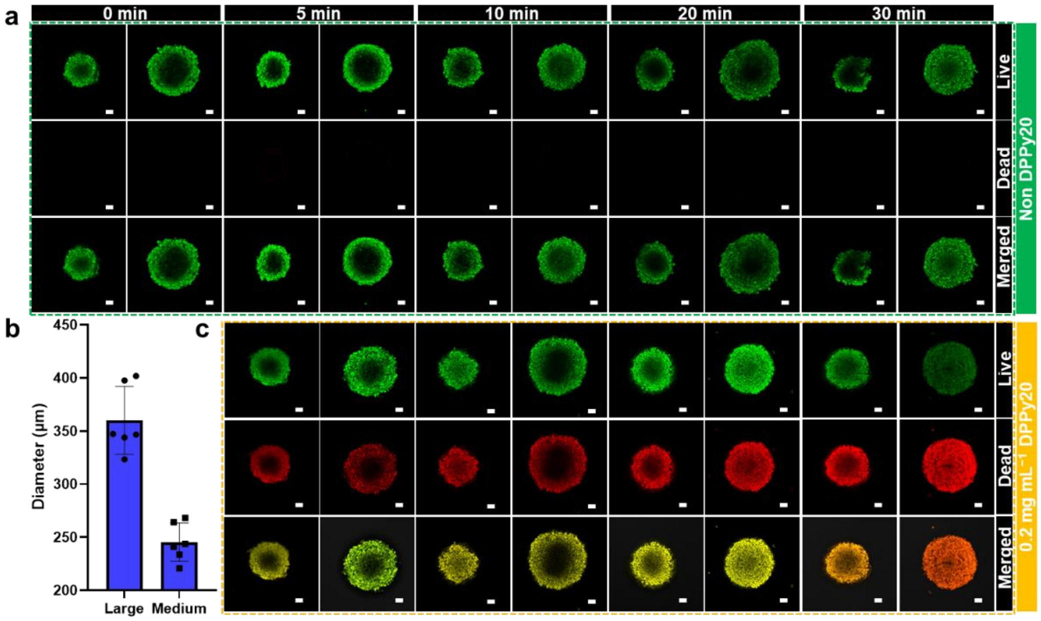

Figure S14. Therapeutic effect mediated by DPPy20 nanosheets toward HeLa spheroids. a, CLSM images of calcein-AM/EthD-1 stained HeLa spheroids only treated with laser irradiation. Medium (245.2 ± 18.1 µm) and large (360.0 ± 32.0 µm) HeLa spheroids were cultured without DPPy20 for 24 h before the laser irradiation. The spheroids were then treated with 808 nm laser irradiation alone (0.5 W cm^{-2}) for 0 min, 5 min, 10 min, 20 min, and 30 min. b, Diameters of medium and large HeLa spheroids prepared using 50 µL and 100 µL cell suspensions (1 × 10^3 cell mL^{-1}). Data shown as mean ± S.D., n = 6. c, CLSM images of calcein-AM/EthD-1 stained HeLa spheroids treated with laser irradiation and DPPy20 exposure. HeLa spheroids were cultured with DPPy20 for 24 h before the laser irradiation. The spheroids were then treated with 808 nm laser irradiation (0.5 W cm^{-2}) for 5 min, 10 min, 20 min, and 30 min. All cells were dyed with calcein-AM (Live) and EthD-1 (Dead). Scale bar, 50 µm.

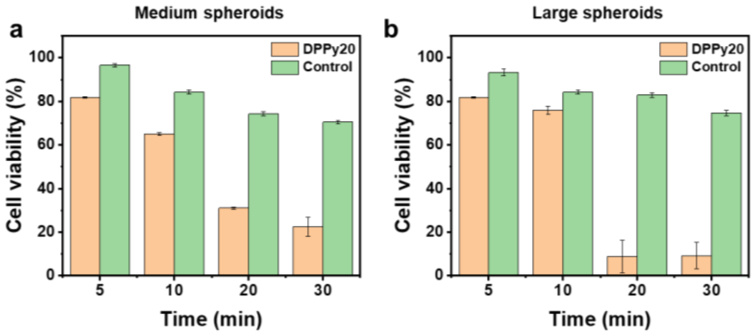

Figure S15. Photothermal therapy efficiency of DPPy20. a, DPPy20 therapeutic performance in medium spheroids after NIR laser treatment (808 nm laser with 0.5 W cm^{-2} in power density) for 5 min, 10 min, 20 min, and 30 min. b, DPPy20 therapeutic performance in large spheroids after NIR laser treatment (808 nm laser with 0.5 W cm^{-2} in power density) for 5 min, 10 min, 20 min, and 30 min. The size of the spheroids was shown in Figure S14b. Data shown as mean ± s.d., N = 3 independent experiments.

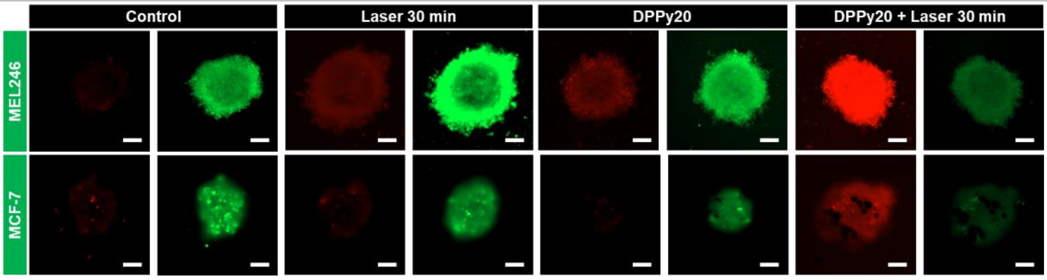

Figure S16. Fluorescence images of MEL246 and MCF-7 spheroids stained with calcein-AM/EthD-1. Freshly prepared spheroids without laser irradiation and DPPy20 treatment were used as control. Spheroids treated with laser irradiation for 30 min alone and DPPy20 for 24 h alone were also imaged. The images were used to show the effect of laser irradiation and DPPy20 on the cell vaibility, repectively. In terms of DPPy20 + Laser 30 min, DPPy20 (0.2 mg mL^{-1}) nanosheets were incubated with the 3D spheroids for 24 h before exposure to 808 nm laser irradiation for 30 min. Green channel: calcein-AM, live cells; Red channel: EthD-1, dead cells. Scale bars, 100 µm.

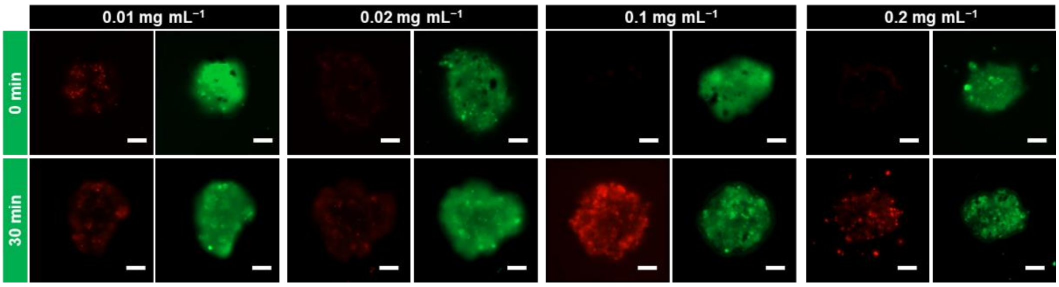

Figure S17. Dose-dependent therapeutic effect mediated by DPPy20 nanosheets toward MCF-7 spheroids with 808 nm laser irradiation (0.5 W cm^{-2}) for 30 min. The spheroids were incubated with DPPy20 at various doses (0.01 mg mL^{-1}, 0.02 mg mL^{-1}, 0.1 mg mL^{-1}, and 0.2 mg mL^{-1}). Fluorescence images of spheroids stained with calcein-AM/EthD-1. Green channel: calcein-AM, live cells; Red channel: EthD-1, dead cells. Scale bars, 100 µm.

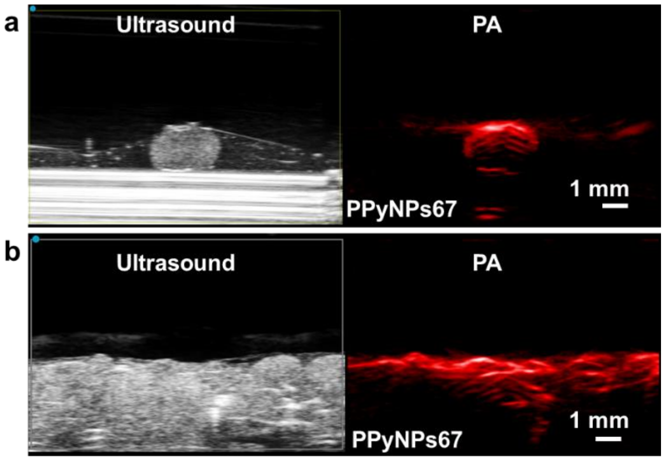

Figure S18. Representative PA images of PPyNPs67 (0.5 mg mL^{-1}) in an alginate sphere phantom (a) and in a mouse (b).

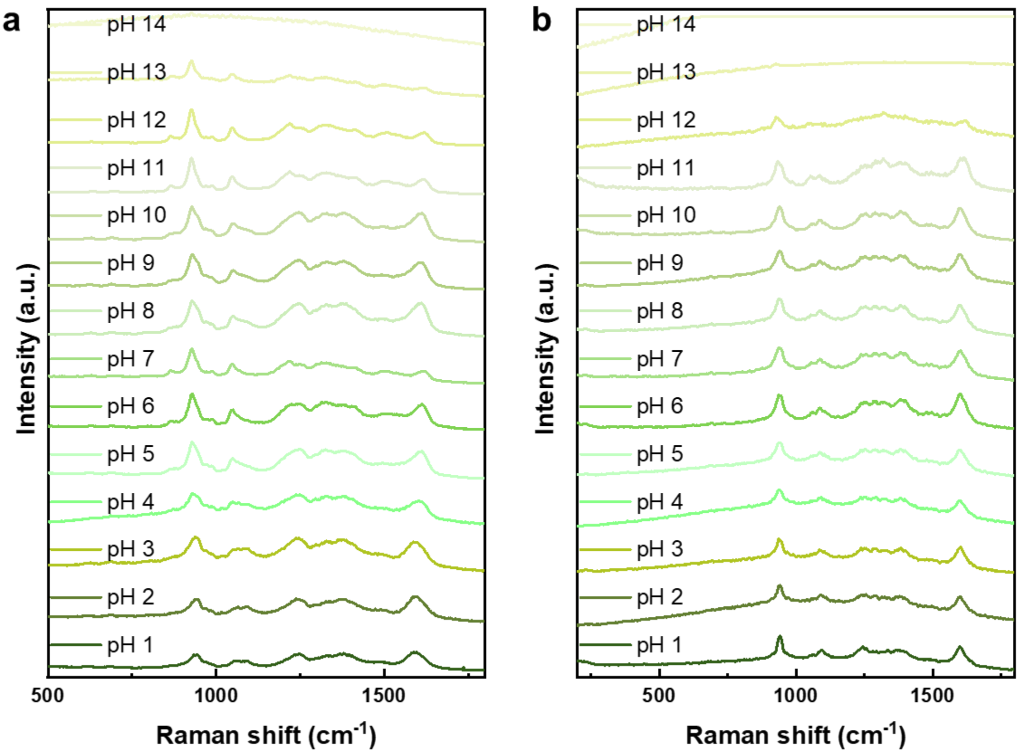

Figure S19. Raman spectra of DPPy20 nanosheets (a) and PPy nanospheres (b) with various pH values. Each line represents the mean of 10 obtained spectra employing a 785 nm laser beam and a charge-coupled device detector with 4 cm^{-1} resolution.

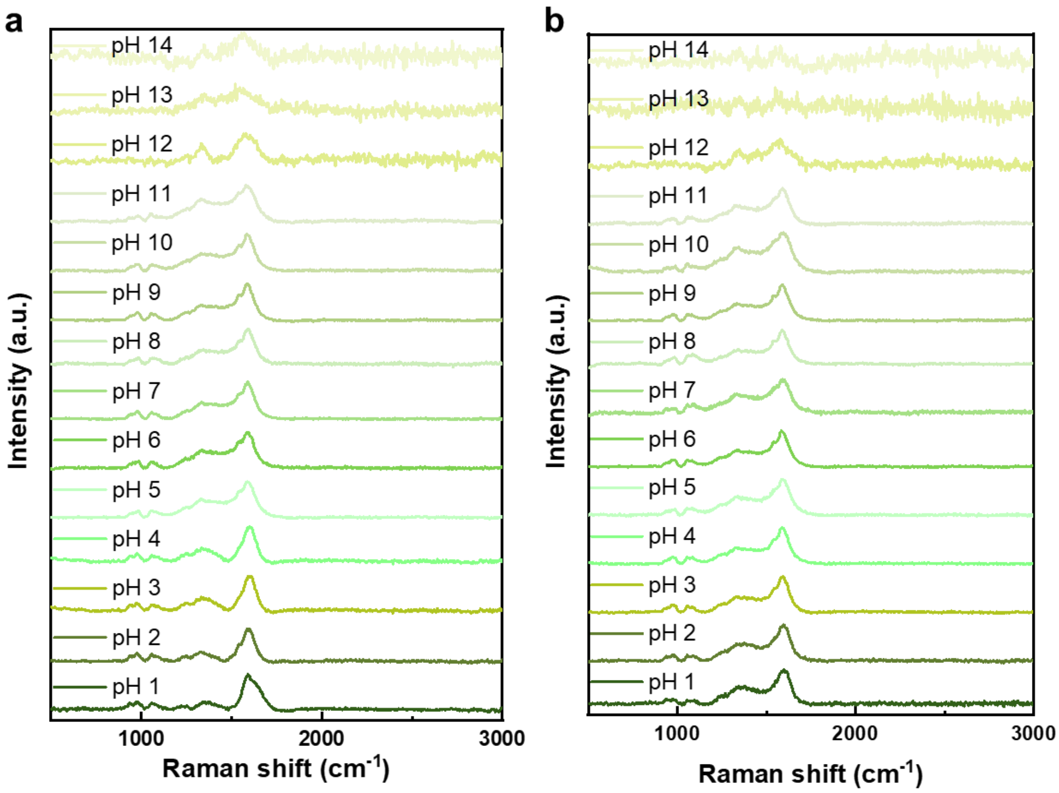

Figure S20. Raman spectra of DPPy20 nanosheets (a) and PPy nanospheres (b) with various pH values. Each line represents the mean of 10 obtained spectra employing a 532 nm laser beam and a charge-coupled device detector with 4 cm^{-1} resolution.

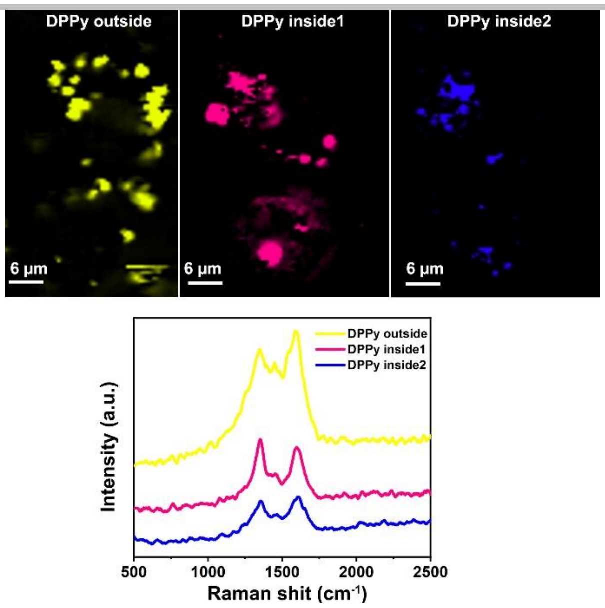

Figure S21. Representative Raman spectra of DPPy20 inside and outside HeLa cells, indicating the pH values of the microenvironment.

References

[1] J. Tabačiarová, M. Mičušík, P. Fedorko, M. Omastová, *Polym. Degrad. Stab.* **2015**, *120*, 392-401.

Author Contributions

H.G. designed the study, prepared and characterized the hybrid nanosheets, interpreted the data, and wrote the paper. E.J.L. helped with PAI imaging and contributed to the manuscript. R.S. helped with data analysis and contributed to the manuscript and aided in study design and discussion. C.L.G. contributed to the manuscript. K.Z. and Y.M. helped with characterizing samples. G.B. helped with cell culture and sample preparation and contributed to the manuscript. D.S. contributed to the *in vivo* studies and edited the manuscript. H.G. performed all Raman experiments and analysis. M.M.S. contributed to study design, scientific discussions, revised the manuscript, and supervised the project. All authors were involved in the discussion and editing of the manuscript.
